# Photoresponsive Photoacid-Macroion Nano-Assemblies

**DOI:** 10.3390/polym12081746

**Published:** 2020-08-05

**Authors:** Alexander Zika, Sarah Bernhardt, Franziska Gröhn

**Affiliations:** Department of Chemistry and Pharmacy & Interdisciplinary Center for Molecular Materials, Friedrich-Alexander Universität Erlangen-Nürnberg, Egerlandstr. 3, D-91058 Erlangen, Germany; alexander.az.zika@fau.de (A.Z.); sarah.bernhardt@fau.de (S.B.)

**Keywords:** electrostatic self-assembly, irradiation, nano-assemblies, nanostructures, polyelectrolyte, photoacid, photoresponsive, supramolecular, switchability

## Abstract

In this study, light-responsive nano-assemblies with light-switchable size based on photoacids are presented. Anionic disulfonated napthol derivates and cationic dendrimer macroions are used as building blocks for electrostatic self-assembly. Nanoparticles are already formed under the exclusion of light as a result of electrostatic interactions. Upon photoexcitation, an excited-state dissociation of the photoacidic hydroxyl group takes place, which leads to a more highly charged linker molecule and, subsequently, to a change in size and structure of the nano-assemblies. The effects of the charge ratio and the concentration on the stability have been examined with absorption spectroscopy and ζ-potential measurements. The influence of the chemical structure of three isomeric photoacids on the size and shape of the nanoscale aggregates has been studied by dynamic light scattering and atomic force microscopy, revealing a direct correlation of the strength of the photoacid with the changes of the assemblies upon irradiation.

## 1. Introduction

In mother nature, the concept of self-assembly is vital for life, as it generates much of the functionality of living cells [1]. It also bears great synthetic potential for the formation of versatile, switchable, and functional nanostructures [2,3,4,5]. Noncovalent interactions can be triggered by external influences, such as the change of pH [6,7,8,9], light irradiation [10,11,12,13,14], thermal activation [15,16,17,18], introduction of a magnetic field [19,20,21], moisture, or redox response [22,23,24,25]. Of high interest are light-responsive systems, for example in the fields of sensors [26,27,28] or therapy [29,30,31], and, thus, it is desirable to explore novel concepts toward light-triggerable self-assembly. Classical approaches use functional groups undergoing photoinduced changes, such as cis–trans isomerization [32,33] and bond cleavage [34,35], which have been extensively studied. The unique capability of photoacids to transfer charges in the electronic excited state opens new perspectives for light-stimuli-responsive supramolecular chemistry [36], which has remained almost unexploited for nano-assemblies so far.

Recently we have used this unique behavior of photoacids to trigger the formation of nano-assemblies from molecularly dissolved building blocks upon light irradiation [37]. In the first model system, this was realized by excited-state proton transfers from a photoacid to a polybase upon photoexcitation, which led to the formation a divalent ionic linker interconnecting the macroions [37]. Fundamentally, the assembly formation in this novel type of responsive systems is based on electrostatic self-assembly, a concept that has been established over the last few years [38,39,40]. In this approach the formation of assemblies is driven by electrostatic interactions in combination with secondary interactions, such as π–π interactions and geometric forces, which leads to well-defined nano-architectures in solution. Hence, the advantage of this concept is the general combination of interactions, instead of relying on specific binding motifs, which makes this approach facile and versatile as compared to other formations of nano-assemblies. A fundamental understanding regarding the interplaying interaction forces was developed [41,42,43,44]. Further, switchable structures based on electrostatic self-assembly were studied [45], in particular, the combination of a polyelectrolyte with a light-switchable molecule, such as azobenzenes [46] or spiropyrane [47], and—in one first concept study—a photoacid [37].

**Scheme 1:**
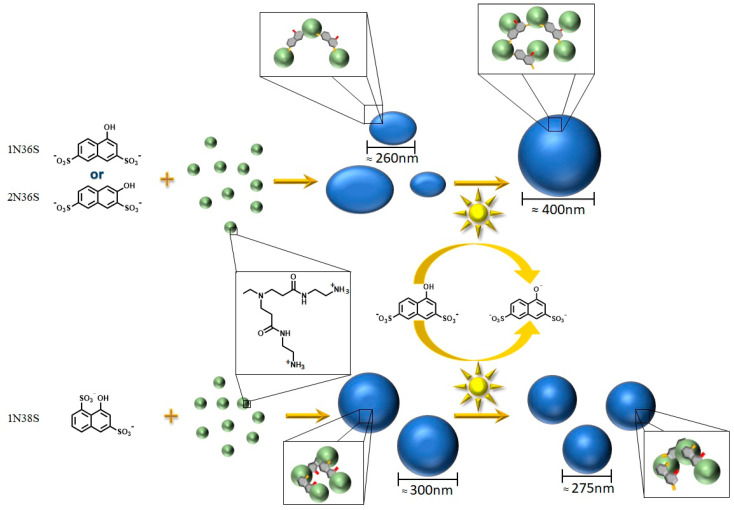
Schematic overview on the assembly formation from a photoacid and a cationic dendrimer. One of the three photoacids is mixed with the G4 dendrimer. The formed nano-assemblies are irradiated. Due to the photoacid’s charge increase, changes in size and structure occur.

With regard to the potential of light-triggerable self-assembly in different fields, it is desirable to establish a system where a photoacid trigger can be used to switch the size and structure of self-assembled entities in solution. As a model system, the divalent anionic photoacid sodium 1-naphthol-3,6-disulfonate was combined with the cationic polyelectrolyte poly(amidoamine) dendrimer of the 4th generation (G4) in aqueous solution (Scheme 1). Due to the two negative charges of the photoacid in the ground state, nano-assemblies are already formed upon combination of the components by electrostatic self-assembly before irradiation. Upon irradiation, these photoacids undergo an increase in acidity of the hydroxyl proton and undergo an excited-state intermolecular dissociation reaction [48,49,50]. This opens the possibility to use the photoinduced creation of more highly charged molecules to change the properties of already existing nano-assemblies.

Photoinduced changes on the nanoscale were detected by dynamic light scattering (DLS), atomic force microscopy (AFM), and ζ-potential measurements. Absorption and emission spectroscopy were used to detect possible photochemical reactions and to elucidate the supramolecular arrangement. Moreover, the position isomerism of the photoacids influences the behavior on the nanoscale.

## 2. Materials and Methods 

The poly(amido amine) dendrimer of generation 4 (PAMAM, G4) was purchased from Dendritech (Midland, MI, USA). The photoacids 1-naphthol-3,6-disulfonate (>90%) and 2-naphthol-2,7-disulfonate (≥95%) were purchased from Sigma Aldrich (Munich, Germany), and the photoacid 1-napthol-3,8-disulfonate (≥95%) was from abcr-GmbH (Karlsruhe, Germany). Deionized water was filtered with two 25 mm syringe filters, which possess a hydrophile membrane consisting of polytetrafluoroethylene with 200 nm pore size.

Prior to sample preparation a stock solution of each chemical was prepared in deionized water and filtered through Millex-LG (Sigma-Aldrich, Munich, Germany) water at a pH = 7.0. The pH was detected with a HI 221 Microprocessor pH meter and adjusted by adding NaOH or HCl (1N, filtered with Millex-LG filter). For the photoacids, the stock solutions were stored under light exclusion. After addition of the photoacid to water, the dendrimer was added to the solution under stirring and light exclusion. The final concentration (c = 1 × 10^−4^ mol/l) of the photoacid was kept constant for every sample. The concentration of the dendrimer varied according to the anticipated charge ratios. To remove dust particles, the samples were centrifuged for five minutes at 3500 rpm.

For UV light irradiation, a 200 W Hg(Xe) Newport Oriel Apex Illuminator (Irvine, CA, USA) with the UV bandpass filter FSQ_UG5 of a wavelength range of 200 nm ≤ λ ≤ 420 nm was used.

For centrifugation, a centrifuge SIGMA 2-16K with a maximum speed of 15300 rpm was used. It was equipped with an angle rotor (no. 12148), which has a capacity of 24 × 1.5 mL and a maximum relative centrifugal force of 24 × 5 g.

DLS was carried out with an instrument equipped with an ALV 5000 correlator with 320 channels (ALV GmbH, Langen, Germany), an ALV CGS 3 goniometer, and a red HeNe laser (λ = 632.8 nm, 20 mW). The samples were measured over a scattering angular range of 30° ≤ θ ≤ 150 ° in steps of 10° for the duration of 50 seconds. Via the Siegert relation, the intensity of the autocorrelation function g^2^(τ) was transferred into the electric field autocorrelation function g^1^(τ). By a regularized inverse Laplace transformation, the electric field autocorrelation function g^1^(τ) was successively transformed into the distribution of relaxation times A(τ). As a result, the apparent diffusion coefficients were calculated by

(1) $$D_{app}=\frac{\Gamma }{q^{2}}$$

The diffusion coefficients were obtained from angular-dependent measurements via extrapolation to zero scattering vector square and via Stokes–Einstein relation the hydrodynamic radii resulted.

Absorption spectra were recorded on a SHIMADZU UV Spectrophotometer (UV-1800) with a slit width of 1.0 nm and a range of 200 nm ≤ λ ≤ 800 nm. The spectra were recorded against air as reference. For all measurements 10 mm quartz cuvettes were used.

Atomic force microscopy (AFM) was performed using NanoWizard 4 from a JPK instrument (Berlin, Germany) operated in tapping mode with a fixed-spring cantilever holder and a USC-F0.3-k0.3-10 ultrashort cantilever with a force constant of 0.3 Nm^−1^. AFM samples were prepared by drop-casting the solution on a freshly cleaved mica substrate. Dendrimer–photoacid samples before and after irradiation were blow-dried after 10 min. The images were analyzed using Gwyddion 2.47. The volumes of the AFM structures have been calculated using a spherical cap as model structure.

ζ-potential measurements were carried out with a Zetasizer Nano ZS analyzer with a 4 mW HeNe laser (λ = 633 nm; Malvern Instruments Ltd., Malvern, U.K.). The solutions were placed in folded capillary cells (DTS 1070). After applying an electric field across the sample solution, the electrophoretic mobility was measured by using the technique of laser Doppler anemometry. By using the Smoluchowski approximation, the ζ-potential was calculated from the electrophoretic mobility. The measurements were performed at 20 °C and repeated three times to gain an average value.

The Spartan’14 software (Wave function Inc., Irvine, CA, USA, 2014) was used for the calculations. Molecular properties and electrostatic potential surfaces were generated with the density functional B3LYP level of theory using 6-31G* basis set in vacuum. All molecules were optimized for the equilibrium geometry with the maxima and minima in the electrostatic potential surface determined. The polyar surface area (PSA) has been calculated taking into account all the polar atoms in the molecule.

## 3. Results

### 3.1. Model System: Electrostatic Self-Assembly with 1-Napthol-3,6-Disulfonate (1N36S)

First, nano-assemblies from dendrimer and the strongest photoacid 1N36S were investigated. Photoacids are molecules, which can undergo an excited state intermolecular proton transfer reaction. In the ground state these molecules are only weak Brønsted acids. Upon photoexcitation, the pKa* value in the excited state decreases, subsequently increasing the acidity of the molecule. The photoacid 1N36S has a pKa = 8.6 and in the ecxited state a pKa* = −2.6 [50]. Photoacid and polyelectrolyte stock solutions were mixed in aqueous solutions of pH = 7 to result in different charge ratios. At pH = 7.0 the dendrimer is known to have 64 positive charges from its protonated primary amine groups [51], while the photoacid has two negative charges due to its sulfonate groups. The charge ratio is defined as the molar concentration of primary dendrimer amine groups z^+^ of the dendrimers divided by the molar concentration of sulfonate groups z^−^ of the photoacids before irradiation.

(2) $$r=\frac{z^{+}\times c\left(G4\;dendrimer\right)}{z^{-}\times c\left(Photoacid\right)}$$

**Figure 1:**
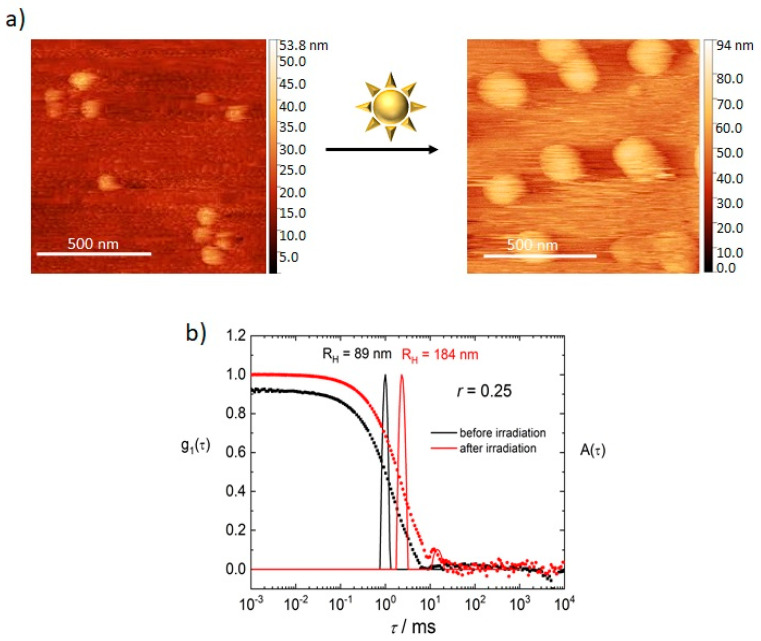
Assembly formation and photoresponse of the dendrimer–photoacid system at a charge ratio of *r* = 0.25: (**a**) AFM height images before (**right**) and after (**left**) irradiation. (**b**) DLS—electric field autocorrelation function g^1^(τ) and distribution of relaxation times A(τ) at a scattering angle of ϴ = 90°.

Figure 1 shows atomic force microscopy (AFM, Figure 1a) and dynamic light scattering (DLS, Figure 1b) results for a sample with a charge ratio *r* = 0.25 before and after UV irradiation. Before irradiation, the narrow size distribution (standard deviation σ = 0.19) from DLS measurements shows that defined nano-assemblies already form before irradiation due to electrostatic interaction of the anionic dye sulfonate groups and cationic dendrimer charges (Figure 1b, black line). Yet the intensity of the scattering is low, indicating a small number of assemblies. 

Upon irradiation, the size of the assemblies yields even more defined nanoparticles (σ = 0.11, Figure 1b, red line). At the same time, the scattering intensity increases by a factor of nearly 1000, which, even considering that the scattered light is proportional to r^6^ (radius to the power of six), indicates the formation of more nano-assemblies. Measurement of the pH-value also showed a decrease from pH = 7.0 to a more acidic pH = 4.3. This shows that the change of the nano-assemblies upon irradiation occurs not only due to the formation of a more highly charged photoacid but also due to the more acidic environment and subsequent partial-protonation of the dendrimer.

The AFM results are in good agreement with the DLS measurements. Before irradiation AFM shows that the sample with a charge ratio of *r* = 0.25 after deposition on a mica surface and drying exhibits particles with a diameter of around d = (130–240) nm. After light exposure, the diameter measured in AFM ranges between d = 200 nm and d = 250 nm. To compare the sizes of the assemblies, the volumes of the nanoparticles were calculated, which were converted into a hypothetical radius of a volume-equivalent spherical particle and compared to the hydrodynamic radius from the DLS. The average radii calculated from the AFM volumes lie around R_AFM_ = 87 nm for the nonirradiated samples and R_AFM_ = 117 nm for the irradiated samples. These results show that the relative size change is nearly the same as observed in DLS. AFM yields smaller sizes because the assemblies are dried after the deposition on the surface. The size decrease upon drying is similar to values reported before, indicating that the structure is swollen in solution and shrinks upon drying [43].

**Figure 2:**
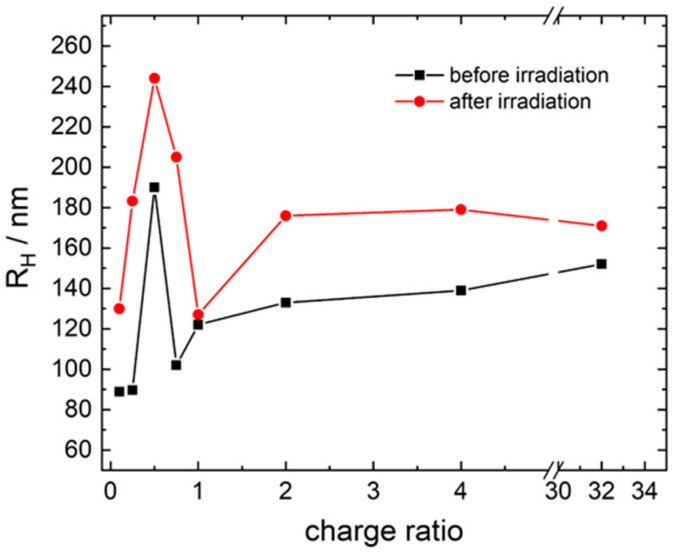
Assembly formation and photoresponse of the dendrimer–photoacid system: DLS—dependency of R_H_ on the charge ratio before and after irradiation.

The dependency of the size and the size response upon irradiation on the charge ratio is considered in Figure 2 and an example of DLS results is also shown in Figure S1. Hydrodynamic radii range from R_H_ = 90 nm to R_H_ = 190 nm before irradiation. Here, at a charge ratio smaller than *r* = 2.0, the DLS measurement shows a narrow distribution, while higher charge ratios show an expressed polydispersity with three or more particle sizes ranging from R_H_ = 2.0 nm to R_H_ = 1000 nm. The peak at the smallest relaxation time corresponding to the smallest particles yields an R_H_ = 2.0 nm. This radius represents the size of a G4 dendrimer showing that free dendrimer molecules coexist with the assemblies. Upon irradiation, DLS reveals a size increase with hydrodynamic radii ranging from R_H_ = 130 to R_H_ = 244 nm, while all particle size distributions become more narrow. The dependency of the assembly sizes on the charge ratio shows the same trend as prior to photoirradiation. Further, at a higher concentration of the building blocks (c(1N36S) = 9.33 × 10^−3^ mol/L) the behavior of the particles is the same (for data see Figures S2 and S3). Although the size of the nano-assemblies for both concentrations is quite similar before the irradiation, upon irradiation the size increases to a R_H_ = 421 nm at the higher concentration, a size nearly twice as large as the lower concentration of c(1N36S) = 1.00 × 10^−4^ mol/L.

**Figure 3:**
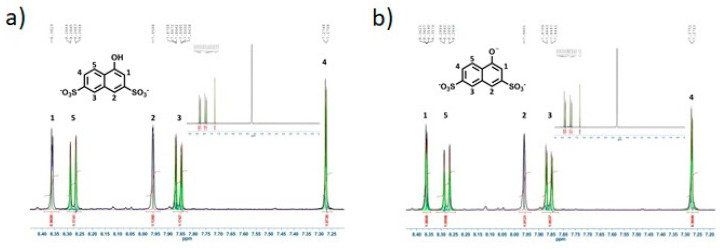
Assembly formation and photoresponse of the dendrimer–photoacid system: ^1^H-NMR of pure 1N36S (**a**) before and (**b**) after irradiation.

To test the possibility of a chemical reaction occurring instead of an excited state intermolecular proton transfer, a solution of 1N36S was measured with ^1^H-NMR before and after irradiation. As can be seen in Figure 3a,b, the NMR spectra of the sample before and after irradiation show the same peaks. This proves that upon photoexcitation the photoacid is not degraded and the effects of irradiation on the assemblies are the result of the photoacidity effect.

**Figure 4:**
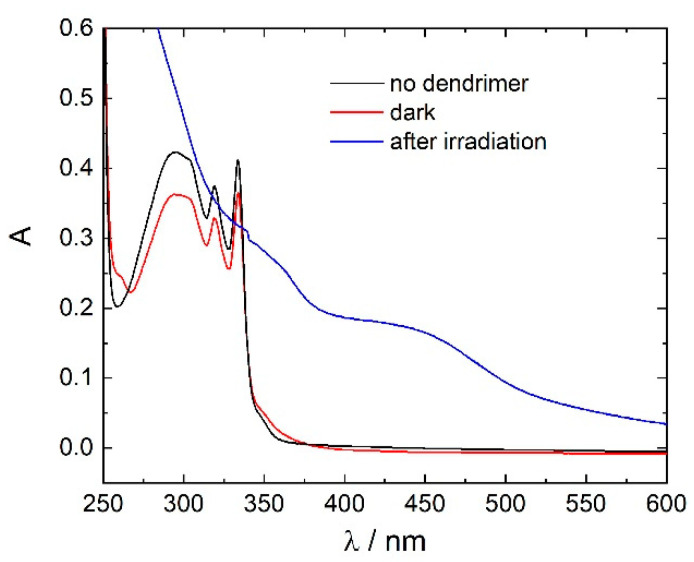
Assembly formation and photoresponse of the dendrimer–photoacid system: UV/Vis absorption spectroscopy of the nano-assemblies at *r* = 0.25 before and after irradiation.

Since degradation through light irradiation has been excluded, UV/Vis spectroscopy can now serve to analyze the supramolecular arrangement. As can be seen in Figure 4, the addition of dendrimer in the dark instantly leads to a slight decrease in intensity of the photoacid band at λ = 300 nm. Upon irradiation, the band at λ = 300 nm is distorted. Next to a newly formed shoulder at λ = 350 nm a new band at λ = 450 nm appears. This dependency can be understood when comparing with the pH dependent measurement of the photoacid only (Figure S4), which reveals that the band at λ = 300 nm corresponds to the protonated photoacid, the band at λ = 350 nm to the unprotonated photoacid, while no band at λ = 450 nm appears in the photoacid only solution in the complete range from 7 ≤ pH ≤ 13. This suggests that the photoacid deprotonates upon irradiation also in the assembly system, and that the new band at λ = 450 nm originates in the new assemblies formed.

**Figure 5:**
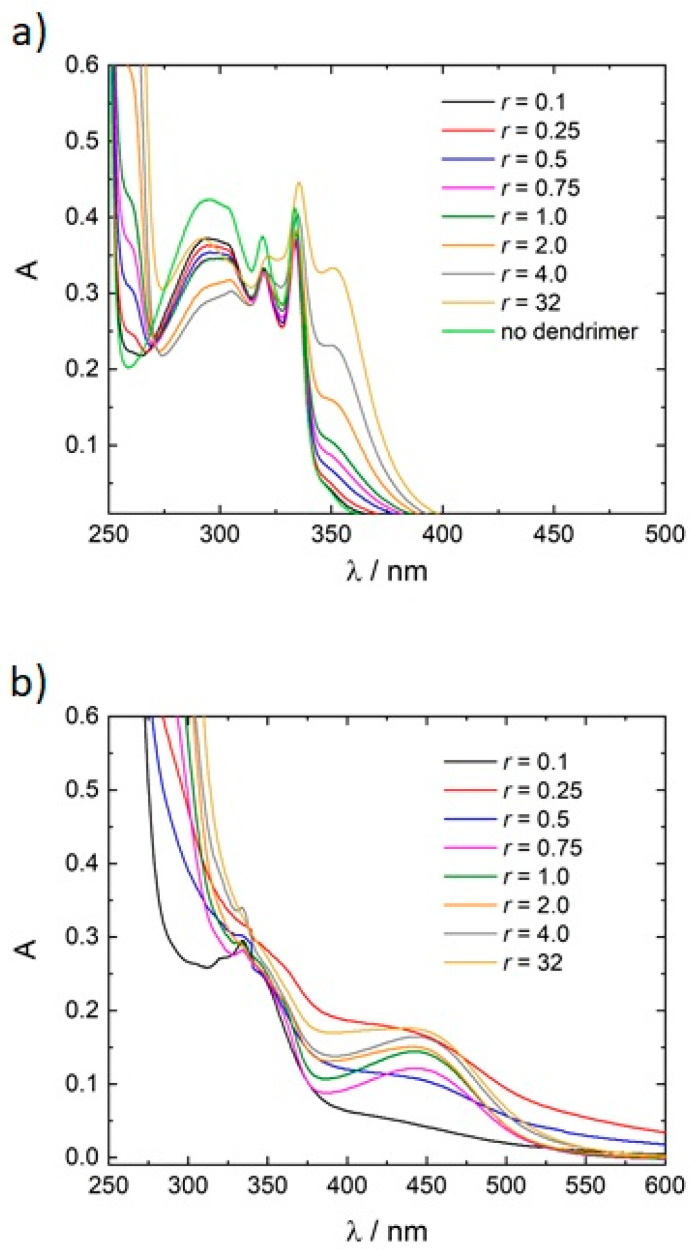
Assembly formation and photoresponse of the dendrimer–photoacid system: UV/Vis absorption spectroscopy—G4/1N36S solution at different charge ratios r (**a**) before and (**b**) after irradiation.

To be able to extract more information on the changes in the absorption spectra, more charge ratios have been analyzed. As shown in Figure 5a, the addition of dendrimer in the dark instantly leads to slight changes of the absorption spectrum in the form of a new band at λ = 352 nm. With increasing concentration of the polyelectrolyte, the new band shows an increase in intensity. As mentioned before, this new band corresponds to the deprotonated species. Correspondingly, the band at λ = 300 nm decreases in intensity. Thus, upon addition of dendrimer, the photoacid becomes more and more deprotonated. Upon irradiation (Figure 5b), the prominent broad band between λ = 300 nm and λ = 340 nm is observed until a charge ratio of *r* = 0.25 is reached and then completely disappears with increasing concentration of the dendrimer. This suggests that at low charge ratios, where there is an excess of photoacid molecules, free 1N36S is still in solution. Furthermore, a new band is formed at λ = 450 nm. At low charge ratios the band is not distinct due to scattering. From a charge ratio of *r* = 0.75 upwards the band becomes more distinct. Since this band was not observed before the excitation without dendrimer, it can be assigned to the formation of nano-assemblies from the photoacid and the dendrimer.

**Figure 6:**
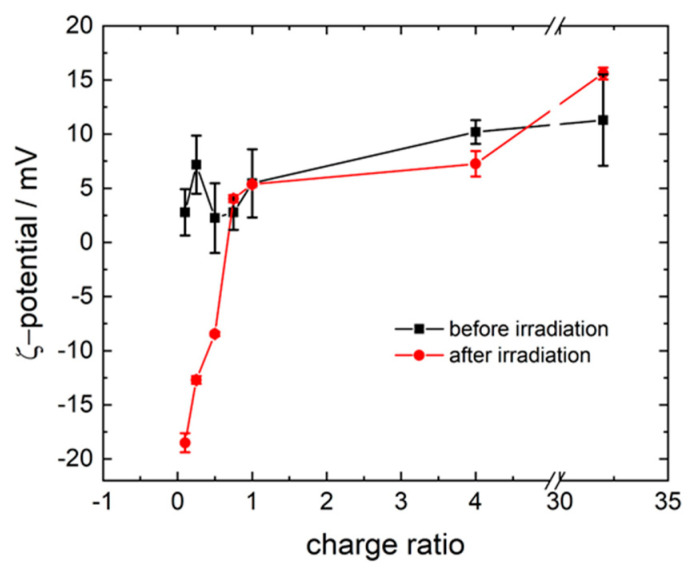
ζ-potential of G4/1N36S solutions: comparison before and after irradiation.

To gain more information on the particle structuring, ζ-potential was measured. As evident in Figure 6, all assemblies before excitation show a positive ζ-potential. With an increasing ratio, the potential rises. This shows that despite the excess of photoacid molecules, and thereby excess of anionic charges in the sample, remarkably the assemblies contain an excess of cationic charges. In addition, the high standard deviation indicates that the number of 1N36S molecules interacting with one dendrimer differs.

Upon photoexcitation, the three lowest charge ratios show a negative ζ-potential, which becomes less negative with the increasing charge ratio. This indicates that upon irradiation, the number of 1N36S molecules per dendrimer molecule increases, leading to an excess of anionic charges. At a ratio of *r* = 0.75, the ζ-potential is the same before the irradiation and starts to be positive again, while it also increases with the increasing charge ratio until saturation is achieved. The standard deviation of the ζ-potential for the irradiated samples is low compared to potentials before irradiation, in agreement with the narrowly distributed size measured in the DLS.

**Scheme 2:**
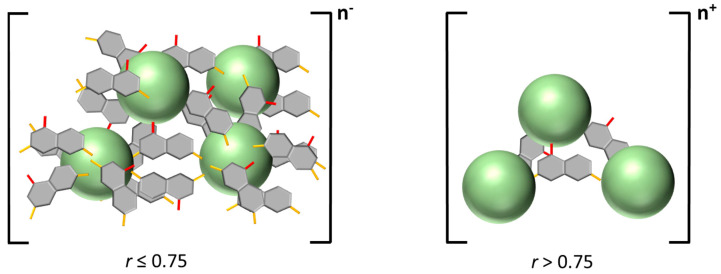
Schematic representation of assemblies of 1N36S with G4 dendrimer. Left: Possible structure for charge ratios smaller than *r* = 0.75. Dendrimer molecules are interconnected by 1N36S molecules. Further 1N36S molecules interact with each other and the dendrimer. Right: Possible structure for charge ratios higher than *r* = 0.75. Dendrimer molecules are interconnected by 1N36S molecules. No further 1N36S molecules interact with the dendrimer.

In a combined consideration of UV/Vis, ^1^H-NMR, AFM, DLS, and ζ-potential results, the following picture is in agreement with the light-induced change, as depicted in Scheme 2: At an excess of anionic charges, the polyelectrolyte is interconnected by 1N36S molecules. Further photoacid molecules can be found interacting with only one dendrimer in singular dendrimer–dye assemblies. Due to the excess of anionic charges, the size of the electrostatically stabilized assemblies is small. With increasing charge ratio, the number of photoacid molecules added per dendrimer decreases, and the size of the assemblies increases. At a charge ratio *r* > 0.75 the dendrimers are still interconnected, but fewer 1N36S molecules are incorporated. Due to the repulsion of the dendrimers, the size decreases again with increasing concentration of the dendrimer.

Thus, electrostatic self-assembly of a cationic dendrimer and a divalent anionic photoacid in aqueous solution occuring in the dark yields assemblies in the R_H_ = 90–190 nm range depending on the charge ratio. Photoexcitation causes a significant increase in photoacid dissociation and, thus, a change in the electrostatic assembly.

### 3.2. Variation of the Photoacid

To gain a deeper understanding on the structure-directing effects, the influence of using three isomeric photoacids—1N36S (as discussed above), 2-naphthol-2,7-disulfonate (2N36S), and 1-naphthol-3,8-disulfonate (1N38S)—has been investigated.

**Figure 7:**
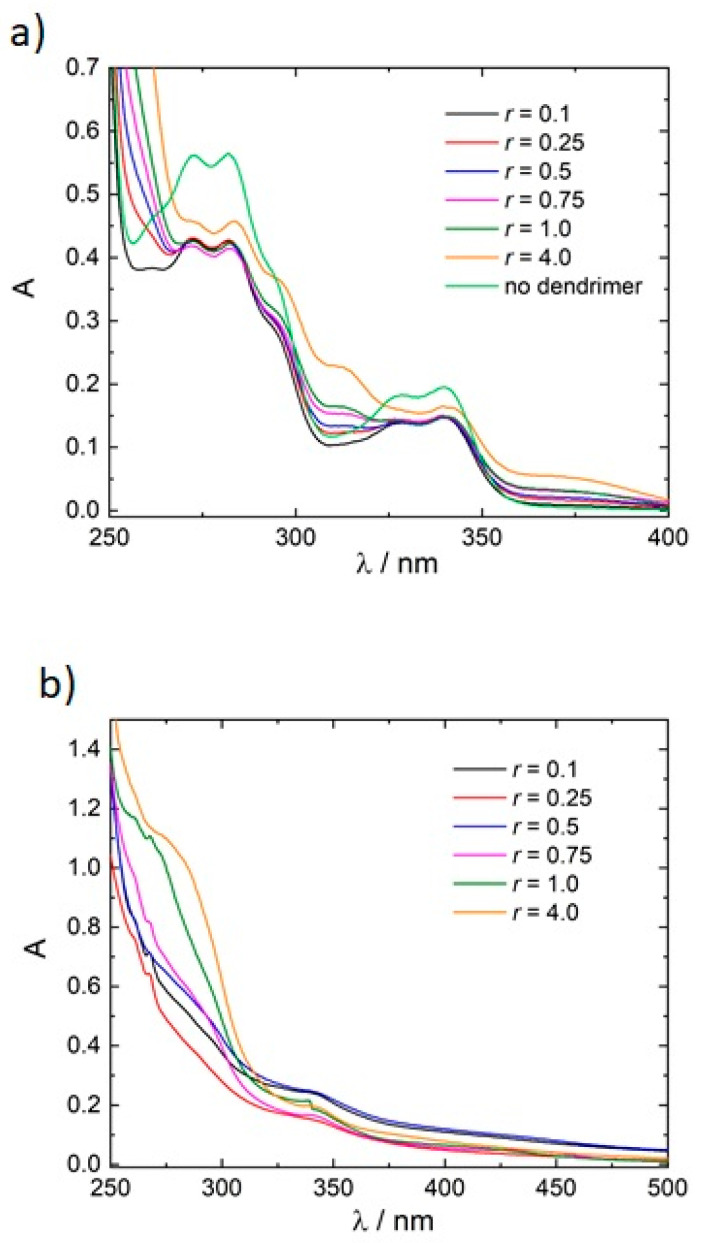
Assembly formation and photoresponse of the dendrimer–photoacid system: UV/Vis absorption spectroscopy—G4/2N36S solution at different charge ratios (**a**) before and (**b**) after irradiation.

For 2N36S, the change of the position of the hydroxyl group weakens the photoacidity. The pKa-value of 2N36S is pKa = 8.7, while in the excited state it is pKa* = 0.7 [52,53]. Next to the different molecular structure, the strength of the photoacid should influence the molecular transformation upon irradiation. To analyze this, absorption spectra before and after irradiation have been measured, as given in Figure 7a,b. With increasing concentration of the dendrimer, the spectra before photoirradiation display a blue shift of two new bands at λ = 310 nm and λ = 380 nm compared to *r* = 0.1. These two bands correspond to the deprotonated form of 2N36S, demonstrating that with increasing concentration of polyelectrolyte more photoacid molecules become deprotonated. This was also observed for 1N36S. The spectra change drastically upon photoexcitation. No distinct bands corresponding to the photoacid molecules can be seen apart from the band at λ = 270 nm and λ = 340 nm. This behavior again is similar to 1N36S, where the prominent band corresponding to 1N36S disappeared upon irradiation and with an increasing charge ratio.

**Figure 8:**
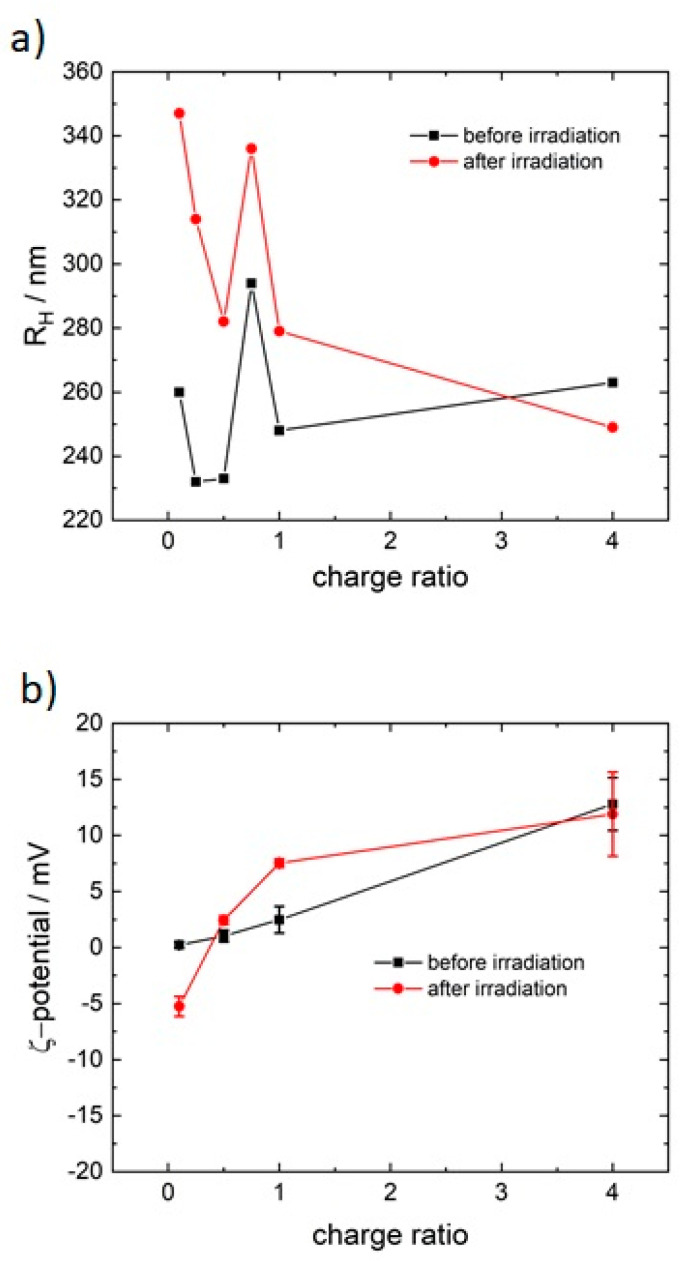
Assembly formation and photoresponse of the dendrimer–photoacid system G4/2N36S: DLS (**a**) dependency of R_H_ on the charge ratio. ζ-potential and (**b**) dependency of ζ-potential on the charge ratio.

To compare the sizes of the assemblies, DLS has been performed before and after irradiation. The autocorrelation function shows that nano-assemblies are present and monodisperse in the dark (see Figure S5). The size of the assemblies before the excitation is around R_H_ = 265 nm; that is, nearly twice as large compared to 1N36S. Upon irradiation the hydrodynamic radius of the particles increases, as given in Figure 8a. The size significantly decreases from the lowest charge ratio to a ratio of *r* = 0.5, which can be understood with the higher concentration of the dendrimer. With the range of R_H_ = 250 nm to R_H_ = 350 nm, the size of the assemblies after irradiation is also higher than the one with 1N36S. Yet, the difference is not as high as before excitation, indicating that the photoirradiation influences 2N36S less than 1N36S. This can be understood with the strength of the photoacidity. The change of position of the hydroxyl group evidently influences the size and the topology, although the behavior as such stays the same.

The ζ-potential is shown in Figure 8b. The assemblies with 2N36S before excitation show exactly the same behavior as 1N36S and exhibit a positive ζ-potential. In general, the scenario and the possible structure of the assemblies are similar to the ones of 1N36S, although the changes upon irradiation are not as drastic. This is understandable given the different strengths of the photoacids, since 1N36S is a much stronger acid in the excited state than 2N36S.

**Figure 9:**
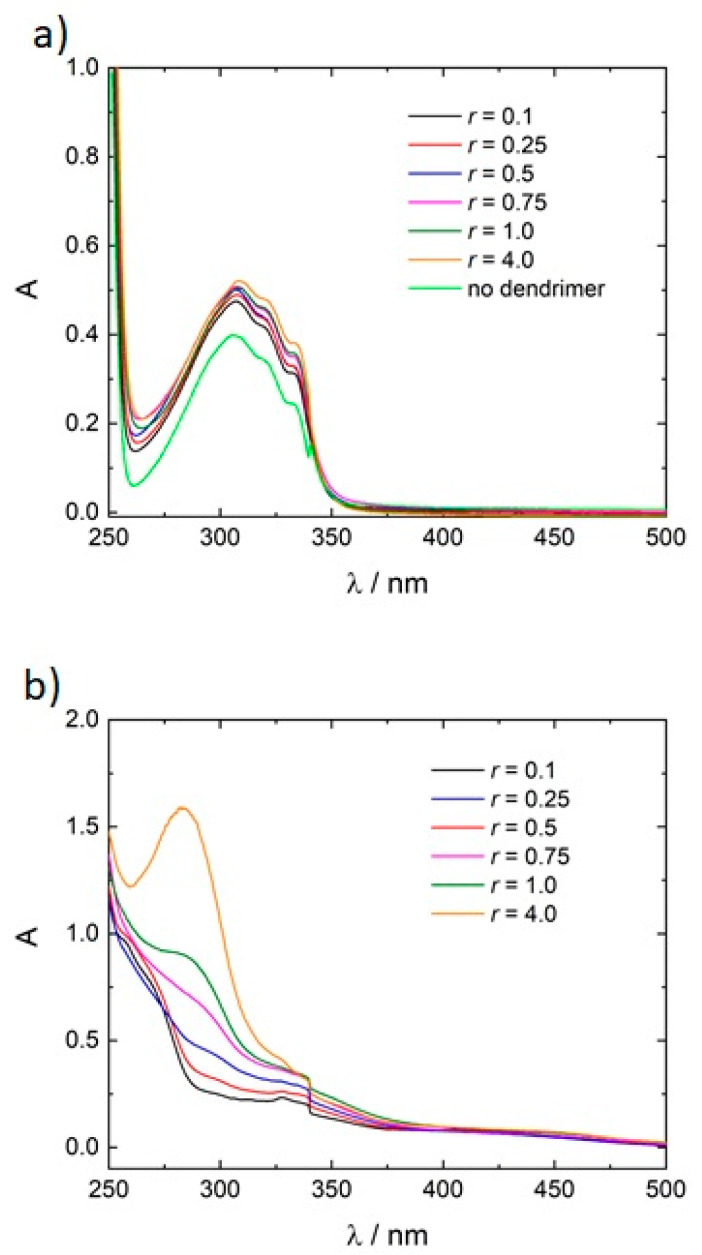
Assembly formation and photoresponse of the dendrimer–photoacid system: UV/Vis absorption spectroscopy—G4/1N38S solution at different charge ratios (**a**) before and (**b**) after irradiation.

For 1-naphthol-3,8-disulfonate (1N38S), instead of the change of the hydroxyl group, the position of one of the two sulfonate groups differs. In Figure 9a, absorption spectroscopy of the nonirradiated samples shows the distinct band at λ = 320 nm, which corresponds to the protonated form of 1N38S. With increasing concentration of the polyelectrolyte, the intensity of the band increases. This indicates the generation of the deprotonated form (as evident from the comparison with the pH dependent spectra given in Figure S6). Only at *r* = 4.0 ratio, the shoulder characteristic of the trivalent anion starts to appear.

Upon photoexcitation, the characteristic band for the 1N38S molecule disappears (Figure 9b). A new band is formed at λ = 290 nm. This corresponds to the free dendrimer in solution. With an increasing charge ratio, more and more dendrimer molecules become unbound. Furthermore, a small band is formed around λ = 450 nm for 1N36S, which is independent of the charge ratio.

**Figure 10:**
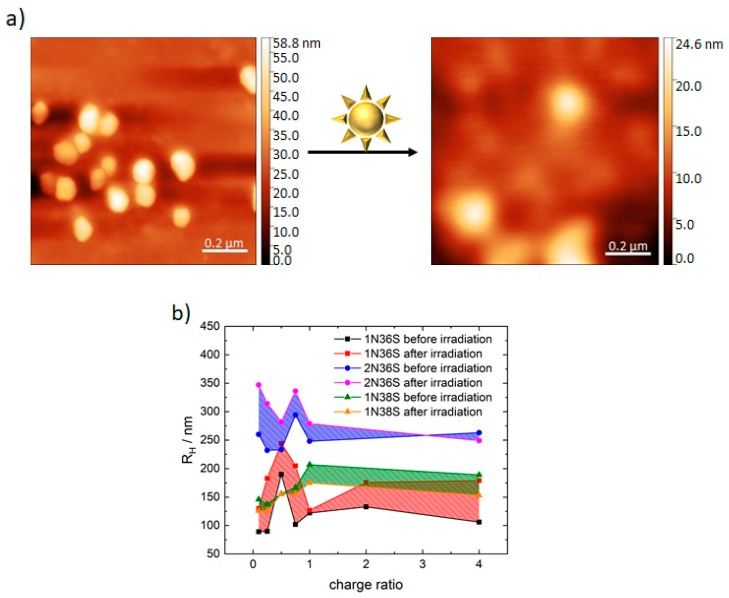
Assembly formation and photoresponse of the dendrimer–photoacid system: (**a**) AFM height images of 1N38S/G4 at *r* = 0.25 before and after irradiation and (**b**) dependency of R_H_ on the charge ratio. Comparison of the three photoacids.

Figure 10 compares the photoacids in terms of AFM (Figure 10 a in comparison to Figure 1) and DLS (Figure 10b, see also Figure S7). Before irradiation, the size at an excess of negative charges is in the range of 138 nm ≤ R_H_ ≤ 167 nm and only increases significantly at a balanced charge ratio or an excess of positive charges. Values are higher than for 1N36S but smaller than for 2N36S. The samples with 1N36S and 2N36S show higher R_H_ for an excess of negative charges, while 1N38S exhibits the opposite. As can be seen in the AFM, the topology of the assemblies is similar to 1N36S. Upon photoexcitation, the particle size increases with increasing charge ratio, although at an excess of positive charges the size decreases to a value of R_H_ = 153 nm. The scattering intensity increases by a factor of five as compared to the nonirradiated samples. Evidently, the position of the sulfonate group greatly influences the assembly size. A possible reason could be that both sulfonate groups are in closer proximity and are placed in the same plane, which leads to repulsion and steric hindrance when associating with the dendrimer. Furthermore, electrostatic repulsion of the negative charges can also play a role.

**Figure 11:**
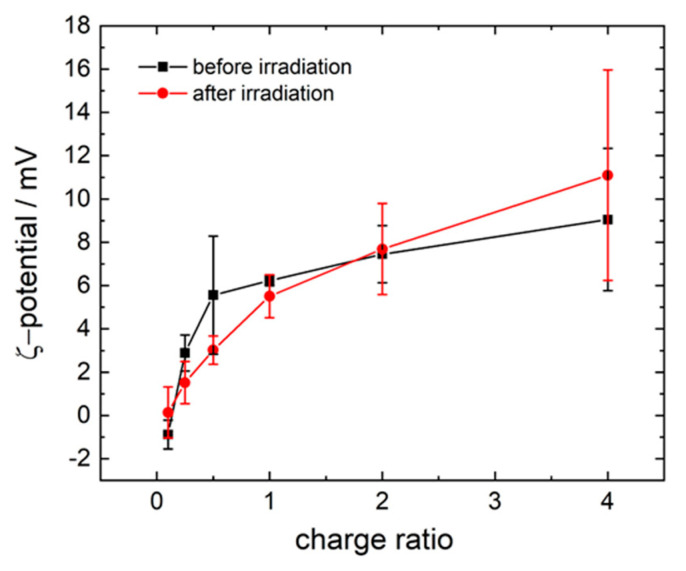
Assembly formation and photoresponse of the dendrimer–photoacid system: ζ-potential dependency on the charge ratio.

ζ-potential measurements are given in Figure 11. Except for the lowest ratios, the assemblies show a positive potential before excitation. With the increasing ratio, the potential rises and reaches a saturation point at a clear excess of positive charges. This demonstrates that, even though there is an excess of photoacid molecules and negative charges in the solution, the assemblies exhibit a positively charged character. This is similar to 1N36S and 2N36S.

Upon photoexcitation, the ζ-potential changes only slightly. The potential of the lowest charge ratio increases compared to the nonirradiated sample and becomes positive and then increases with increasing charge ratio. In comparison to the nonirradiated samples, the potential decreases except for the lowest and highest ratio. The changes of the potential are so small, indicating that only small changes occur in the structure. This is contrary to the expected strength of the photoacid.

### 3.3. Polar Surface Area of the Photoacid Molecules

**Figure 12:**
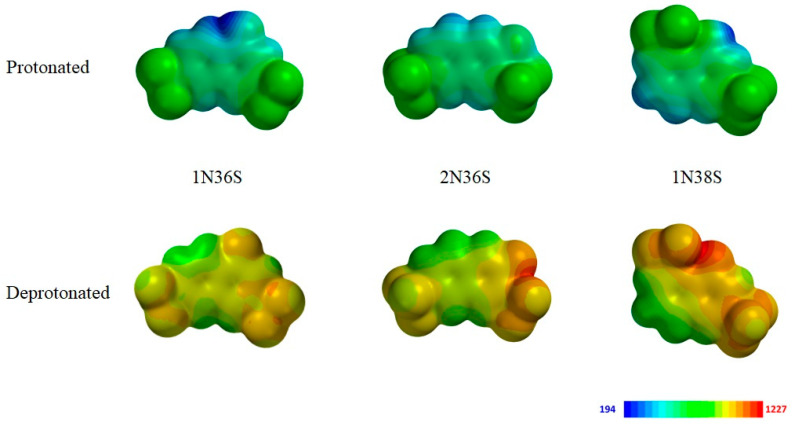
Electrostatic potential at the molecular surface for the photoacid molecules. Top: protonated hydroxyl group. Bottom: deprotonated hydroxyl group.

Previously we have investigated how molecular building-block properties encode nanoparticle size and shape in electrostatic self-assembly in solution. For the electrostatic self-assembly of (nonswitchable) aromatic anionic dye molecules with cationic G4 dendrimers through a combination of ionic and dye–dye interaction, a structure-directing effect of the molecular building blocks’ polar surface area (PSA) on the nanoscale assembly features was found [42]. The PSA is a molecular property based on the electrostatic potential, which again is the three-dimensional charge distribution of the molecule. It results from the polar groups in the molecule and takes the substituents into account more explicitly than the polarizability that refers to the molecule as a whole. It was found that the PSA correlates with the interaction of the molecules, in particular, the mutual dye–dye interaction in the form of a dye–dye electrostatic repulsion contribution. Hence, the lower the PSA the better the dye molecules can interact with each other. The ΔG, as well as the ΔH/ΔS ratio of the association, again determined size and shape on the nanoscale. To understand the different behavior and to clarify the role of π–π interaction in the nano-assembly formation studied herein, the polar surface area (PSA) of the photoacid molecules has been calculated. In Figure 12, a visual representation of the electrostatic potential is given.

Before irradiation, the photoacid molecules all exhibit a similar electrostatic potential. The oxygen of the hydroxyl group withdraws electron density and at the same time reveals the protic character of the hydrogen. Upon irradiation and the subsequent deprotonation of the hydroxyl group, the electrostatic potential becomes more negative, equivalent to a more electron-rich system.

**Figure 13:**
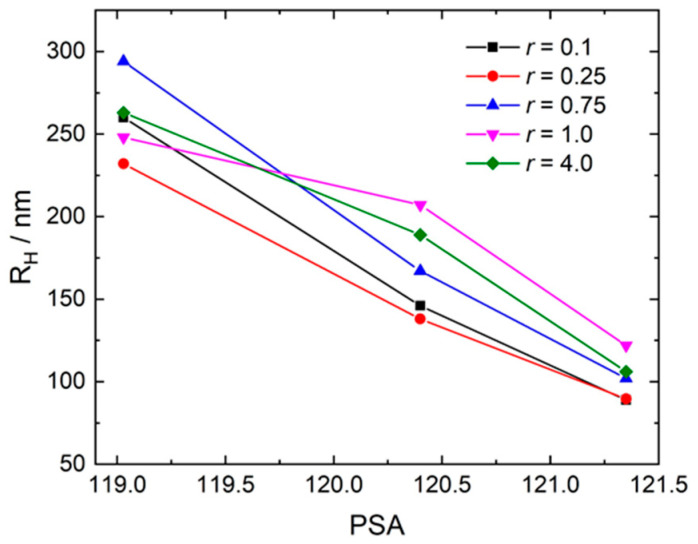
Polar surface area dependency on R_H_ at different charge ratios before irradiation.

In Table 1, the PSA values based on the electrostatic potential are given. Before irradiation, the photoacid 1N36S has the highest PSA, and 2N36S is the lowest. It is likely that, in analogy to the previous study, the lower the PSA the stronger the mutual dye–dye interaction, which occurs through the π–π interaction of the molecule cores and is modified in its strength by the substituents. In that case, 1N36S will have the weakest dye–dye interaction, while 2N36S will have the strongest one. Indeed, this relates to the size of the particles before irradiation. Thus, it can be concluded that the assembly size before irradiation depends on the strength of the photoacid molecules’ π–π interaction. This is especially the case for an excess of negative charges, as can be seen in Figure 13.

Upon irradiation the PSA increases for 1N36S and 2N36S. In contrast, for 1N38S, the PSA stays nearly constant, while at the same time no change of the nano-assembly size is found at an excess of negative charges for this photoacid. The highest change of PSA is evident for 1N36S, which also shows the highest change in nanoscale structure. Yet the increase of the PSA for 1N36S and 2N36S indicates that the size increase after irradiation is not due to stronger dye–dye interactions but rather to the higher charge of the photoacid molecules and, thereby, stronger attractive electrostatic interaction with the dendrimer. This is also in accordance with the changes of the ζ-potential and the changes in scattering intensity. Thus, the different magnitude of changes upon irradiation can be understood with the strength of the photoacid and with the fraction of molecules undergoing the intramolecular proton transfer reaction. 

Hence, the strength of the growth of the nano-assemblies after irradiation can be understood with a more highly charged photoacid and the stronger affinity to the dendrimer. The change of the sulfonate group position has a larger impact. Before irradiation, the difference in size can also be explained with the stronger dye–dye interaction. Upon irradiation, the particle size is nearly constant and only changes for the assemblies at an excess of positive charges. Possible reasons may be the close proximity of the negative charges and steric hindrance.

## 4. Conclusions

In this study, a new water-soluble light-switchable system was developed, which is based on electrostatic self-assembly of a polyelectrolyte and a photoacid. The structural transformation of the nano-assemblies upon irradiation is based on the formation of a more highly charged building block molecule upon photoexcitation of the photoacid, causing a higher degree of dissociation in the excited state. This system bears potential for providing insight into the principle and for establishing applications of photoacid-based assemblies. The concept introduced here may be especially promising for photocatalysis, where the assemblies can either serve as a nanoscale template or be used in combination with another photosensitizer, and as a delivery system in which the structural changes of the assembly provide a desirable transformable platform for triggerable transport.

## Figures and Tables

**Table 1:** Electrostatic parameter of the different photoacids.

Photoacid	Charge	PSA [Å^2^]
1N36S	Protonated	121.4
	Deprotonated	123.6
2N36S	Protonated	119.0
	Deprotonated	121.7
1N38S	Protonated	120.4
	Deprotonated	120.2

## Supplementary Materials

The following are available online at https://www.mdpi.com/2073-4360/12/8/1746/s1: Figure S1: Assembly formation and photoresponse of the dendrimer-1N36S system at r = 4.0. DLS—electric field autocorrelation function g^1^(τ) and distribution of relaxation times A(τ) at a scattering angle of ϴ = 90°. Figure S2: Assembly formation and photoresponse of the dendrimer-1N36S system at a higher concentration at r = 0.5. DLS—electric field autocorrelation function g^1^(τ) and distribution of relaxation times A(τ) at a scattering angle of ϴ = 90°. Figure S3: Assembly formation and photoresponse of the dendrimer-1N36S system at higher concentration (c(1N36S) = 9.33 × 10^−3^ mol/L). DLS—dependency of R_H_ on the charge ratio. Figure S4: UV/Vis spectroscopy of the pH-dependency of 1N36S in solution. Figure S5: Assembly formation and photoresponse of the dendrimer-2N36S system at r = 0.1. DLS—electric field autocorrelation function g^1^(τ) and distribution of relaxation times A(τ) at a scattering angle of ϴ = 90°. Figure S6: UV/Vis spectroscopy of the pH dependency of 1N38S in solution. Figure S7: Assembly formation and photoresponse of the dendrimer-1N38S system at r = 0.1. DLS—electric field autocorrelation function g^1^(τ) and distribution of relaxation times A(τ) at a scattering angle of ϴ = 90°.
