# Supplementary material for: Photoresponsive Photoacid-Macroion Nano-Assemblies
Source: Polymers (Basel). 2020 Aug 5;12(8):1746. doi: 10.3390/polym12081746 (PMC7464814; doi:10.3390/polym12081746)
Supplement: Supplementary file 1 [file polymers-12-01746-s001.pdf]

polymers

MDPI

Article

Photoresponsive Photoacid-Macroion Nano-Assemblies

Alexander Zika, Sarah Bernhardt and Franziska Gröhn*

Department of Chemistry and Pharmacy & Interdisciplinary Center for Molecular Materials, Friedrich-Alexander Universität Erlangen-Nürnberg, Egerlandstr. 3, D-91058 Erlangen. alexander.az.zika@fau.de (A.Z); sarah.bernhardt@fau.de (S.B)

* Correspondence: franziska.groehn@fau.de

Received: 21 June 2020; Accepted: 31 July 2020; Published: date

Supporting Information

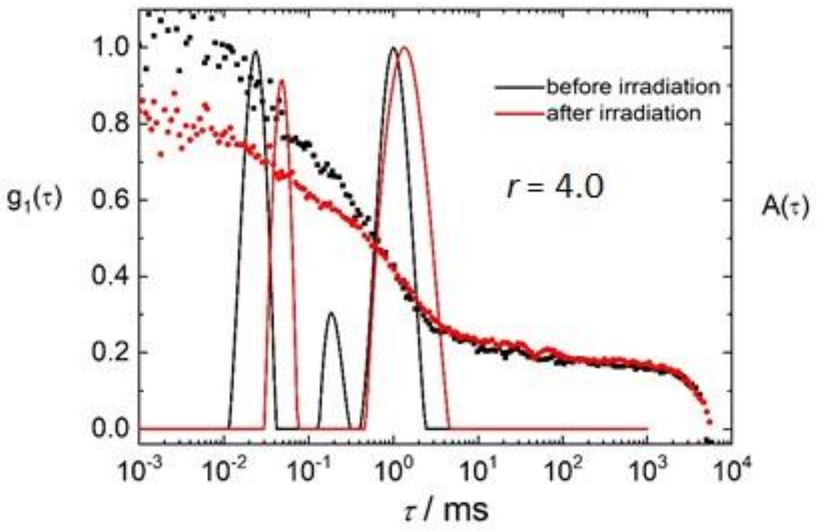

Figure S1. Assembly formation and photoresponse of the dendrimer-1N36S system at r = 4.0. DLS; electric field autocorrelation function $g^1(\tau)$ and distribution of relaxation times $A(\tau)$ at a scattering angle of $\Theta = 90°$.

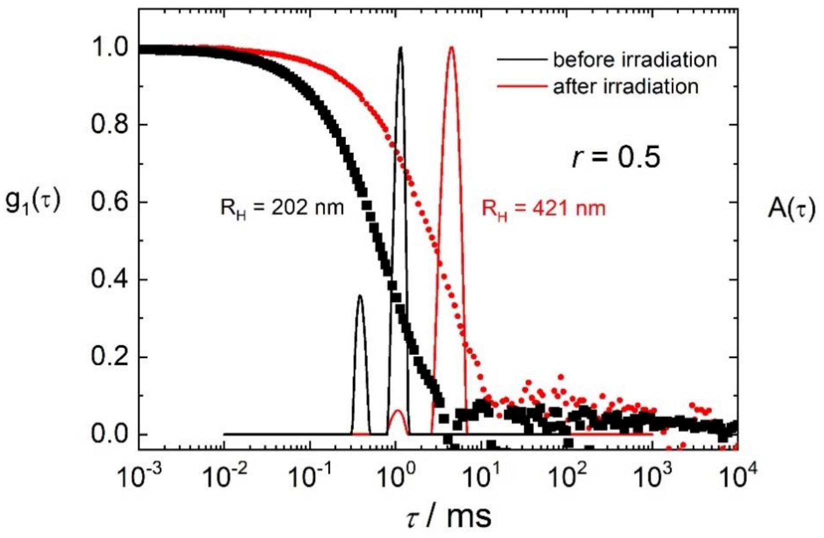

Figure S2. Assembly formation and photoresponse of the dendrimer-1N36S system at higher concentration at r = 0.5. DLS; electric field autocorrelation function $g^1(\tau)$ and distribution of relaxation times $A(\tau)$ at a scattering angle of $\Theta = 90°$.

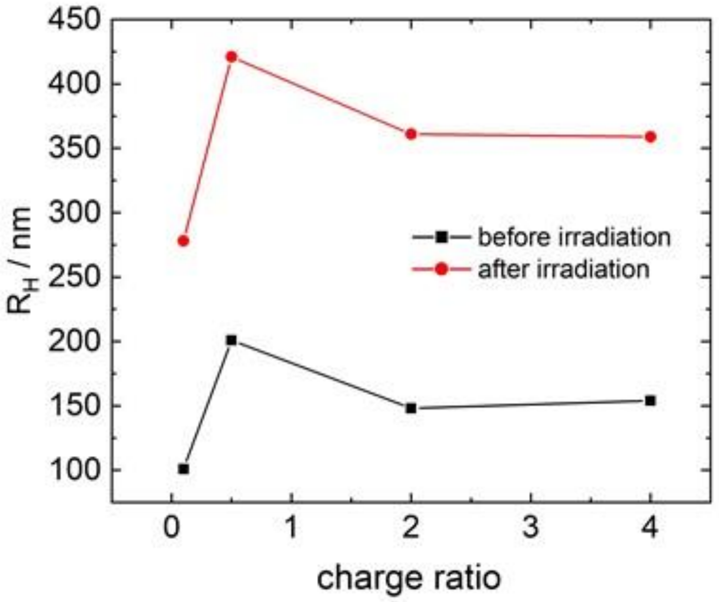

Figure S3. Assembly formation and photoresponse of the dendrimer-1N36S system at higher concentration (c(1N36S) = $9.33 \cdot 10^{-3}$ mol/L). DLS; dependency of R_H on the charge ratio.

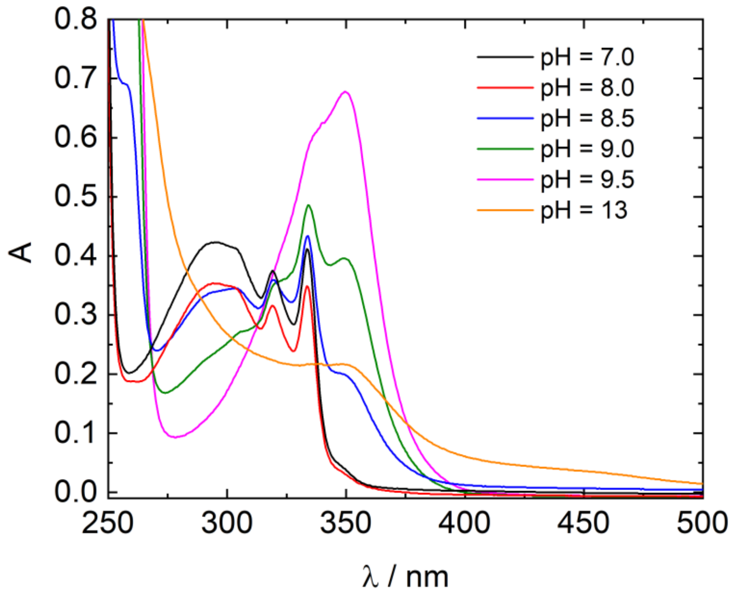

Figure S4. UV/Vis spectroscopy of the pH-dependency of 1N36S in solution.

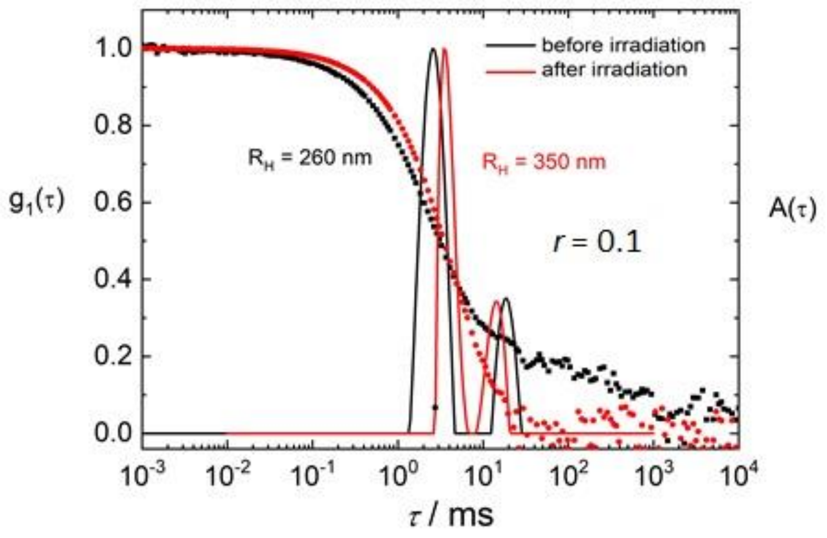

Figure S5. Assembly formation and photoresponse of the dendrimer-2N36S system at r = 0.1. DLS; electric field autocorrelation function $g^1(\tau)$ and distribution of relaxation times $A(\tau)$ at a scattering angle of $\Theta = 90°$.

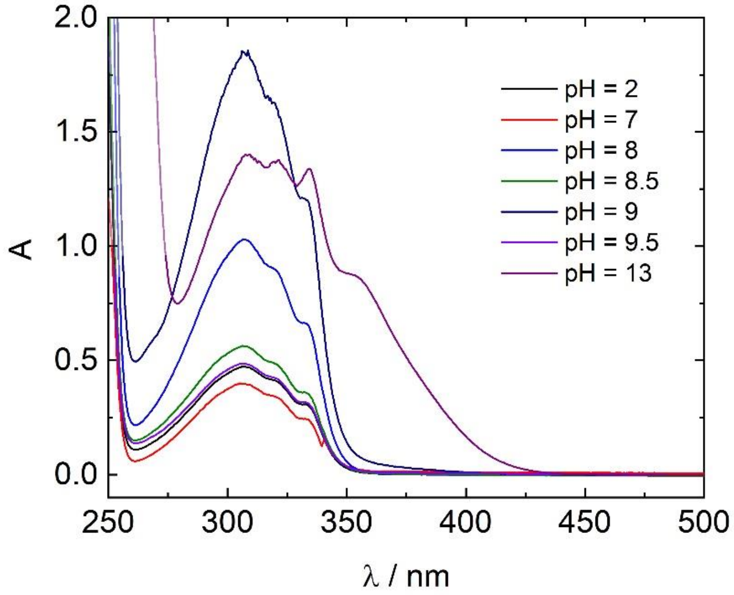

Figure S6. UV/Vis spectroscopy of the pH-dependency of 1N38S in solution.

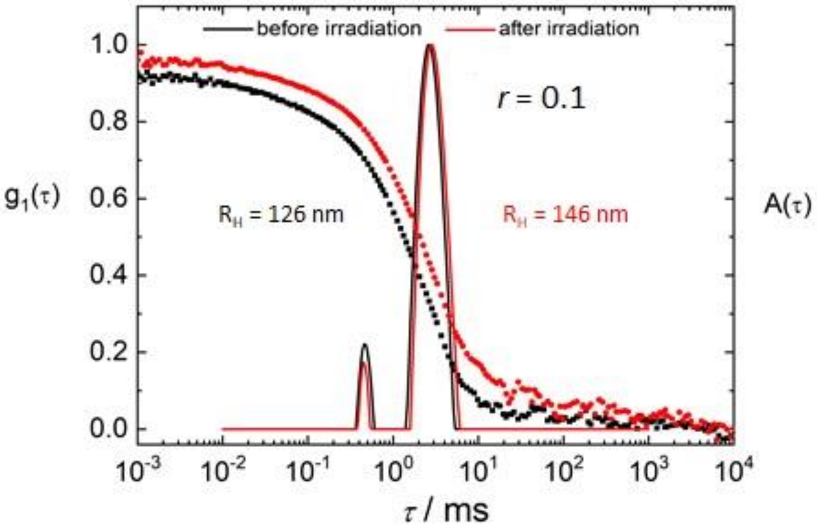

Figure S7. Assembly formation and photoresponse of the dendrimer-1N38S system at r = 0.1. DLS; electric field autocorrelation function $g^1(\tau)$ and distribution of relaxation times $A(\tau)$ at a scattering angle of $\Theta = 90°$.

© 2020 by the authors. Submitted for possible open access publication under the terms and conditions of the Creative Commons Attribution (CC BY) license (http://creativecommons.org/licenses/by/4.0/).
